# Stellate Ganglion Block for the Management of Cannabis Use Disorder: A Case Series

**DOI:** 10.7759/cureus.105898

**Published:** 2026-03-26

**Authors:** N Charan, Varsha Karanth, Shruti Niraj, G Niraj

**Affiliations:** 1 Pain Medicine and Palliative Care, Sri Madhusudan Sai Institute of Medical Sciences & Research, Chikkaballapur, IND; 2 Psychiatry, Sri Madhusudan Sai Institute of Medical Sciences & Research, Chikkaballapur, IND; 3 Psychology and Palliative Care, Sri Madhusudan Sai Institute of Medical Sciences & Research, Chikkaballapur, IND; 4 Pain Medicine and Anaesthesia, Sri Madhusudan Sai Institute of Medical Sciences & Research, Chikkaballapur, IND

**Keywords:** anxiety, autonomic dysfunction, cannabis use disorder, cannabis withdrawal syndrome, stellate ganglion block, sympathetic overactivity

## Abstract

Currently, there are no approved or consistently effective pharmacological treatments for managing cannabis use disorder. Three patients diagnosed with cannabis use disorder and presenting with cannabis withdrawal syndrome were assessed by an interdisciplinary team. These patients were treated with two-level dual stellate ganglion blocks. Validated patient-reported outcome measures were completed. Urine cannabis levels were measured at the four-week follow-up. At one-, two-, and four-week reviews, all three patients reported reduced withdrawal symptoms. Urine cannabis tests were negative at the four-week review. Limitations include a small cohort of patients, absence of a control arm, and possible placebo effects. Stellate ganglion block could have a role in the management of cannabis use disorder and cannabis withdrawal syndrome.

## Introduction

Cannabis use disorder (CUD) is a distinct clinical entity characterized by persistent cannabis use despite adverse effects on social functioning and the physical or mental health of the user or others [1]. The Diagnostic and Statistical Manual of Mental Disorders (DSM)-5 codes the severity of CUD as mild (presence of two to three symptoms), moderate (presence of four to five symptoms), or severe (presence of six or more symptoms) [2]. The prevalence of CUD is higher in users with a history of daily cannabis use and polysubstance use [3]. Abrupt cessation of cannabis products containing tetrahydrocannabinol (THC) results in withdrawal symptoms in a significant proportion of users [2-4]. Frequently reported symptoms include anxiety, irritability, anger, aggression, sleep disturbances, low mood, and reduced appetite. These symptoms develop within 24 to 48 hours of abstinence, peak within 6 days, and last from one to three weeks [1]. Relapse is very common, and the symptoms associated with cessation strongly contribute to relapse [5]. Symptoms such as anxiety, anger, irritability, and low mood can adversely impact interpersonal relationships and work-related productivity [5]. This cohort is more likely to use other substances, including alcohol and nicotine [6]. The pharmacologic treatment options for CUD remain limited, with no approved agents and modest efficacy demonstrated to date [7]. As a result, CUD is often undertreated, especially in rural India.

In patients with cannabis withdrawal, neurochemical changes in the limbic system have been observed, similar to those seen in withdrawal from other substances [4]. The neurobiology suggests dysautonomia with elevated noradrenergic signaling [8]. Stellate ganglion block has been shown to attenuate sympathetic overactivity. We have previously reported on the effectiveness of SGB in managing alcohol as well as opioid withdrawal symptoms [9]. We present three patients with heavy cannabis use (>3 g/day) who presented with severe CUD following abrupt cessation and were successfully managed with SGB.

## Case presentation

Patients presenting with cannabis withdrawal were initially assessed by an interdisciplinary team (IDT) at Sri Madhusudan Sai Institute of Medical Sciences & Research, a tertiary care medical college in South India. The IDT comprised psychiatrists, clinical psychologists, palliative care physicians, and pain medicine physicians. Patients were offered a trial of ultrasound-guided dual two-level SGB. Dual SGB is a standard intervention for treatment-resistant mental disorders, including substance use withdrawal, at our center [9,10]. The interdisciplinary team prospectively follows patients within a longitudinal service evaluation to assess satisfaction with team-based management. The project is registered with the Institutional Clinical and Medical Audit Committee (SMSIMSR/CMAC/17C). Patient satisfaction was assessed using a five-point Likert scale (very satisfied, satisfied, neutral, dissatisfied, very dissatisfied) (Table 1). Informed written consent was obtained. Additional written consent was obtained for the use of their de-identified data for publication in a peer-reviewed journal.

**Table 1:** Demographics and patient characteristics ADHD: attention deficit hyperactivity disorder, BMI: body mass index, MASH: metabolic dysfunction-associated steatohepatitis, THC: tetrahydrocannabinol.

Case	Age (years)	Gender	BMI	Duration of cannabis use (years)	Comorbidity		Urine THC at 4-week review	Patient satisfaction at 4 weeks
Case 1	31	M	27	10	Alcohol misuse, MASH	Negative		Very satisfied
Case 2	26	M	26	15	ADHD, alcohol misuse		Negative	Very satisfied
Case 3	29	M	25	10	Panic attacks		Negative	Very satisfied

Two SGBs were performed with an interval of 24-48 hours between the blocks. Patients completed validated outcome measures, including the Cannabis Withdrawal Scale (CWS) and Generalized Anxiety Scale (GAD-7), weekly for four weeks post-intervention. SGB was performed using real-time ultrasound guidance with an in-plane technique under standard cardiovascular monitoring in an outpatient setting [10,11]. Patients were premedicated with intravenous midazolam 1 mg for sedation and to increase the seizure threshold. An anterolateral approach was used after carefully scanning the neck anatomy with Doppler imaging to identify common structures and vascular anomalies. The needle was visualized under real-time ultrasound guidance through the sternocleidomastoid muscle until the needle tip penetrated the ventral fascia of longus coli, dorsal to the common carotid artery. The injectate consisted of 5 mL of 0.25% bupivacaine, 1 mL of 2% lidocaine, 20 mg of depot methylprednisolone, and 6 mL of distilled water. The block was first performed at the cervical C6 level (7 mL), followed by the block at the C4 level (5 mL). The contralateral blocks were performed within 48 hours [11].

Case 1

A 31-year-old male presented to the IDT with symptoms consistent with severe cannabis withdrawal (DSM-5 code F12.20). He had been smoking 5-6 joints daily for over five years. In addition, the patient reported active cigarette smoking and moderate alcohol use. Several previous attempts at cannabis cessation were reported, all complicated by severe withdrawal symptoms. In response, alcohol intake increased, and cannabis use was resumed within 24 hours. Following a heated argument with his partner, he had abruptly discontinued cannabis. The withdrawal symptoms at presentation included severe anxiety, irritability, insomnia, headache, photophobia, restlessness, anger and aggression toward his partner, low mood, and reduced appetite. He reported significant functional impairment at both home and work. On examination, he was restless, his heart rate was 120 beats per minute, and his blood pressure was 144/98 mm Hg. Investigations revealed elevated bilirubin and liver enzymes, with grade 2 fibrosis and fatty liver on ultrasound liver elastography (FibroScan). The CWS score was 153/190, and the GAD-7 score was 21/21.

He underwent dual two-level SGB with an interval of 48 hours between the blocks. At a 24-hour review following the right SGB, he reported a significant improvement in withdrawal symptoms. Headaches, vivid dreams, and photophobia persisted for six days. At the four-week review, he reported abstinence from both cannabis and alcohol. Urine THC was negative at four weeks.

Case 2

A 26-year-old male presented to the IDT for evaluation of severe cannabis withdrawal symptoms after more than ten years of daily cannabis use, averaging three to four joints per day (DSM-5 code F12.20). He reported polysubstance use, including daily alcohol consumption (>750 mL/day) and nicotine smoking. The patient had made multiple unsuccessful attempts to discontinue cannabis use, including a three-month stay at a rehabilitation facility. He also reported significant low mood since a relationship breakdown over a year ago (Patient Health Questionnaire, PHQ-9 = 22). A diagnosis of attention deficit hyperactivity disorder (ADHD) had been established in childhood, and he had trialed multiple medications as well as psychotherapy without benefit. He reported impaired functioning at work, insomnia, loss of appetite, nightmares, and significant anger issues at both work and home.

Following dual two-level SGB, he reported a substantial reduction in anxiety, anger, irritability, restlessness, and insomnia, which persisted during weekly review for four weeks (Table 2). He reported abstinence from cannabis and alcohol at the 4-week review. Urine THC testing was negative at four weeks.

Case 3

A 30-year-old male presented to the IDT with cannabis use disorder (DSM-5 code F12.20). He reported cannabis dependence for 10 years and was smoking 5-6 joints daily. There was a history of weekly alcohol binge drinking (1000-1500 mL) for 15 years. In addition, he was smoking 10-12 cigarettes daily. The patient began using cannabis to manage severe anxiety and panic symptoms. He had previously undergone pharmacologic and psychotherapeutic treatment for anxiety at another center and declined further medication trials. He had smoked cannabis the previous morning and reported moderate withdrawal symptoms. He was extremely keen to discontinue cannabis use and had over five unsuccessful attempts in the past. He underwent dual two-level SGB and was followed up weekly for four weeks. He reported mild withdrawal symptoms during the weekly reviews. At the four-week review, he reported abstinence from both cannabis and alcohol. Urine THC at the four-week review was negative.

**Table 2:** Patient-reported outcomes at baseline and post intervention. CWS: cannabis withdrawal scale, GAD-7: Generalized Anxiety Disorder scale.

Case	Baseline GAD-7	4-week GAD-7	Baseline CWS	1-week CWS	2-week CWS	3-week CWS	4-week CWS
Case 1	21	10	153	64	40	25	37
Case 2	20	8	130	35	21	26	30
Case 3	16	9	113	41	29	20	17

## Discussion

This case series describes the successful management of three patients with severe cannabis use disorder presenting with major withdrawal symptoms and treated with dual two-level stellate ganglion blocks. Currently, pharmacotherapy has shown modest efficacy in the management of cannabis use disorder [8]. The development of safe and effective therapy for CUD is an important unmet public health need [12]. To the best of our knowledge, this is the first report to describe the benefits of dual two-level SGB in managing cannabis withdrawal in patients with severe CUD.

Cannabis is the most widely used illegal drug, with over 244 million current users worldwide and an estimated 31 million current users in India [13,14]. Although there are few risks related to cannabis withdrawal, the greatest risks include relapse and an increased risk of polysubstance use. The patients in this series reported heavy cannabis use and multiple unsuccessful attempts at abstinence, in addition to polysubstance use. The primary motivation to discontinue cannabis use was the inability to function at work. None of the patients received concurrent psychological intervention during the four-week period post-SGB. At the four-week review, all patients reported complete abstinence from cannabis use and alcohol consumption. They reported reduced withdrawal symptoms during the weekly review. Urine THC levels were negative at the end of four weeks in all three patients.

Cannabis withdrawal is clinically relevant because it substantially increases the likelihood of relapse. In addition, concurrent mental ill health and polysubstance use could precipitate complicated withdrawal [1,15]. The comorbidity of cannabis use with a psychiatric disorder is linked to more severe CUD and reduced responsiveness to treatment [15]. Case 2 was diagnosed with coexistent ADHD and reported the use of other substances. The heavy polysubstance use resulted in significant behavioral issues, inability to maintain gainful employment, family discord, and insomnia. At the four-week review, the patient reported marked improvement in anxiety and sleep, and a return to gainful employment.

The risk of negative consequences with the use of high-potency and large quantities of cannabis is well documented. Acute cannabis consumption is also associated with an increased risk of motor vehicle crashes, especially fatal collisions. Chronic effects of cannabis use include mood disorders, exacerbation of psychotic disorders in vulnerable people, cannabis use disorders, withdrawal syndrome, neurocognitive impairments, and cardiovascular and respiratory diseases [16]. Higher levels of cannabis use are consistently associated with an increased risk of psychosis, and this dose-response relationship supports a causal influence [17]. In addition, cannabis use has been associated with affective disorders, with the odds of having an affective disorder being 3.8 times higher in individuals with a diagnosis of CUD [18]. There is a two-fold risk of generalized anxiety disorder in individuals with lifetime cannabis dependence [19]. Case 3 commenced cannabis use to manage symptoms of severe anxiety and panic. Previous attempts at abstinence were unsuccessful due to severe anxiety triggered by withdrawal. At the four-week review, he reported a reduction in anxiety and an absence of panic attacks.

Addiction involves neurobiological stress and sympathetic overactivity. In addition, withdrawal states cause excess activation of central stress pathways [20]. By targeting the sympathetic circuits involved in addiction and withdrawal, SGB can mitigate central stress pathways and reduce craving and dysphoria [20,21]. Based on our experience in treating patients with substance use presenting in withdrawal, we recommend a two-level dual SGB early in the management of acute withdrawal. SGB acts as a treatment catalyst for further psychological interventions, which are key in enabling long-term abstinence in this cohort. Two-level dual SGB appears to provide stronger and more durable dampening of sympathetic overactivity when compared to single-level dual SGB [9,11]. The block is first performed at the cervical C6 level, followed by the C4 level. The total volume used is 12 mL. All precautions required for a single-level SGB are mandated for a two-level SGB. Long-term effects are observed after the use of SGB with short-acting local anesthetic agents, possibly due to attenuation of central sensitization in the sympathetic nervous system. The addition of depot methylprednisolone is likely to enhance the durability of the block [9]. SGB has been reported to result in inhibition of regional as well as systemic sympathetic overactivity [20].

This report has several important limitations, including its observational design, small patient cohort, and limited follow-up. In addition, there is a possibility that any improvement may have been coincidental. However, there is an urgent need for innovative strategies to manage CUD. Cannabis use can act as a gateway for polysubstance use, as observed in this cohort [22].

## Conclusions

In conclusion, dual two-level stellate ganglion blocks could have a role in the management of cannabis use disorder by mitigating cannabis withdrawal symptoms. SGB has shown effectiveness in alleviating various mental health disorders, including anxiety, enhancing sleep quality, and treating withdrawal symptoms. We recommend definitive studies to confirm these early observations.
